# Prospective controlled randomized trial on prevention of postoperative abdominal adhesions by Icodextrin 4% solution after laparotomic operation for small bowel obstruction caused by adherences [POPA study: Prevention of Postoperative Adhesions on behalf of the World Society of Emergency Surgery]

**DOI:** 10.1186/1745-6215-9-74

**Published:** 2008-12-18

**Authors:** Fausto Catena, Luca Ansaloni, Augusto Lauro, Giorgio Ercolani, Luigi D'Alessandro, Antonio Pinna

**Affiliations:** 1 Transplant, General and Emergency Surgery DPT St Orsola-Malpighi University Hospital Via Massarenti 9, 40138 Bologna, Italy

## Abstract

**Background:**

Adhesive small intestine occlusion [ASIO] is an important cause of hospital admission placing a substantial burden on healthcare systems worldwide. Often times, ASIO is associated with significant morbidity and mortality.

Icodextrin 4% solution [Adept, Shire Pharmaceuticals, UK] is a high-molecular-weight a-1,4 glucose polymer that is approved in Europe for use as an intra-operative lavage and a post-operative instillate to reduce the occurrence of post-surgery intra-abdominal adhesions.

There are no randomized trials on the use of this solution to prevent adhesions after ASIO operation in current medical literature.

The current clinical study evaluates the safety and effectiveness of Icodextrin 4% for decreasing the incidence, extent, and severity of adhesions in patients after abdominal surgery for ASIO.

**Design:**

The study project is a prospective, randomized controlled investigation performed in the Department of Transplant, General and Emergency Surgery of St. Orsola-Malpighi University Hospital [Bologna, Italy]. The study is designed and conducted in compliance with the principles of Good Clinical Practice regulations.

The study compares the results of Icodextrin 4% against a control group who does not receive anti-adhesion treatment. This randomized study uses a double-blind procedure to evaluate efficacy end points. In other words, designated third party individuals who are unaware of the treatment assigned to the patients to assess adhesion formation.

**Trial Registration Number:**

ISRCTN22061989

Prospective controlled randomized trial on Prevention of Postoperative Abdominal Adhesions by Icodextrin 4% solution after laparotomic operation for small bowel obstruction caused by adherences

[POPA study: Prevention of Postoperative Adhesions]

## Introduction

Adhesive small intestine occlusion [ASIO] is an important cause of hospital admission placing a substantial burden on healthcare systems worldwide. Often times, ASIO is associated with significant morbidity and mortality. Postoperative adhesions account for > 40 percent of all cases of intestinal obstruction, with 60 to 70 percent of those involving the small bowel [1]. Of patients who require multiple abdominal operation, 30 to 44 percent have adhesion-related intestinal obstruction [2]. For small-bowel obstruction, the incidence rises to 65 to 75 percent [3]. Mortality rates range from 3 percent for simple intestinal obstructions to 30 percent when the bowel becomes necrotic or perforated [3].

The overall recurrence rate for patients who underwent one operation for ASIO is 18% after 10 years and 29% at 30 years. For patients admitted several times for ASIO, the relative risk of recurring ASIO increased in relation to the number of prior ASIO episodes. The cumulative recurrence rate reached 81% for patients with 4 or more ASIO admissions [4]. In the US, adhesiolysis is responsible for > 300,000 hospitalizations annually, accounting for nearly 850,000 days of inpatient care and $1.3 billion in hospitalization and surgical expenditures [5].

An increasing number of adhesion-reduction agents, in the form of site-specific and broad-coverage barriers and solutions, are becoming available to surgical teams to complement optimal surgical techniques. Icodextrin 4% solution [Adept, Shire Pharmaceuticals, UK] is a high-molecular-weight a-1,4 glucose polymer that is approved in Europe for use as an intra-operative lavage and a post-operative instillate to reduce the occurrence of post-surgery intra-abdominal adhesions [6]. The icodextrin colloid is absorbed slowly, resulting in the retention of the fluid within the peritoneal cavity for more than 4 days. The solution reduces adhesions by a process of hydroflotation, keeping the peritoneal organs and tissues apart during the critical post-surgery period when the patient is at greatest risk of adhesion formation [7]. 1 Icodextrin has an extensive safety profile and has been used as a 7.5% solution in continuous ambulatory peritoneal dialysis [CAPD] for > 50,000 patient-years. In addition, preclinical and preliminary clinical studies have demonstrated the safety and efficacy of Icodextrin 4% solution in the reduction of adhesion formation following abdominopelvic surgery [8].

There are no randomized trials on the use of this solution to prevent adhesions after ASIO operation currently present in the medical literature.

The current clinical study evaluates the safety and effectiveness of Icodextrin 4% for decreasing the incidence, extent, and severity of adhesions in patients after abdominal surgery for ASIO.

## Methods

The project is a prospective, randomized controlled investigation performed in the Department of Transplant, General and Emergency Surgery of St. Orsola-Malpighi University Hospital [Bologna, Italy]. The study is designed and conducted in compliance with the principles of Good Clinical Practice regulations.

A computer-generated schedule creates the randomized assignments for the subject and control groups and the results are sealed in numbered envelopes. Participation in the study is requested after diagnosing small bowel obstruction caused by adhesions and determining if the patient fulfills the inclusion criteria [subjects who elect to undergo the surgical laparotomy]. Accordingly to the protocol, the patient signs a document indicating his or her consent and the responsible surgeon discloses the envelope.

If after opening the sealed envelope containing the assignments, the patient declines to enter the trial, the case is excluded from the study [all dropped-out patients are reported].

The responsible surgeon will record the patient's name [and number].

Each group has a sample size of 88 patients [176 patients for the whole study].

All randomized patients [Intention to Treat population] are included in the analysis.

Sample size has been calculated to reach a confidence level of 95% with a power of 80%.

A sample size of 88 patients each group [65 analyzable] is calculated supposing that the ASIO patients [first, second, third or fourth operation for ASIO] have a mean risk of 50% to develop a further episode of ASIO and this risk can be decreased by 25% [from 50% to 25%] with the use of Icodextrin 4%

Inclusion criteria are:

Adult patients [> 18 years]

Submitted to laparotomic surgical procedures for ASIO

Clinical and radiological evidence of adhesive small intestine obstruction

ASA I-III patients

Informed consent

Exclusion criteria

Intrabdominal cancer

Peritoneal contamination

IBD

Positive history of radiotherapy

Patients with an intra-operative findings of different pathology will be excluded from the study

Preoperative data include patient demographics, comorbid conditions [genitourinary, cardiac, pulmonary, gastrointestinal, renal, or rheumatologic] and a detailed history of previous occlusions and surgical procedures.

Decompression with a nasogastric tube is carried out in order to calculate the average nasogastric tube output of each patient [total amount of drainage/duration]. For patients administered Gastrografin before surgery, only the output before the procedure is considered. Intravenous fluid therapy is performed.

Plain abdomen X-rays are done and maximal small intestine diameter is calculated. Duration of symptoms before admission and number of previous operations are also evaluated.

Subjects with ASIO who elect to undergo the surgical laparotomy are enrolled in the study; written informed consent is obtained from each individual and then they are randomized into the study groups. Laparotomic surgical procedure is carried out and existing abdominal cavity adhesions are documented. Subjects are submitted to adhesiolysis with bowel resection if necessary with or without anastomosis. The first group receive traditional treatment [control group] whereas the second group is treated with Icodextrin 4% before abdomen closure. The use of irrigants during surgery is not allowed. Peritoneal contamination is evaluated with cultures.

Per protocol the abdominal fascia is closed with a running PDS suture and the skin is closed with sutures or skin staples.

Only one abdominal drainage in allowed in case of bowel resection and it has to be removed 7 days after the operation.

In case of bowel leakage, the patient will no longer be included in the study results.

For all patients, perioperative parameters are recorded, including blood loss, total length of the midline incision, method of anastomosis, method and timing of incision openings and closures, corticosteroid use. Operative wounds are classified as clean, clean contaminated, and contaminated as described by Schwartz [9].

**Figure 1:**
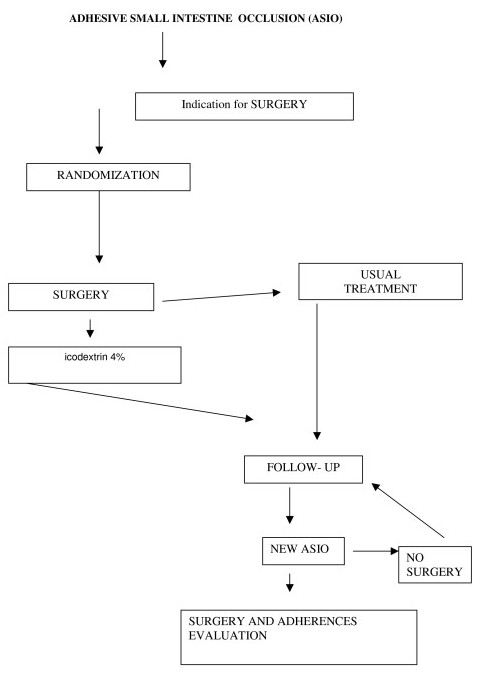


Morbility, mortality and postoperative stay are registered. A flow chart showing the protocol is illustrated in figure 1.

The patients are followed-up for 5 years.

In case of reoperation for ASIO the procedure is carried out by a third party blinded to the patient's previous antiadhesive treatment: this surgeon evaluates incidence, location, severity, and extent of adhesions. The incidence of adhesions is assigned a severity score of 0 [no adhesions], 1 [filmy thickness, avascular], 2 [moderate thickness, limited vascularity], or 3 [dense thickness, vascularized].

Adverse events [AEs] are collected for the duration of the study, beginning at the time of randomization.

AEs are identified and described by the primary investigators.

In the control group, patients will be treated as in our daily surgical practice.

In the informed consent form, patients receive all the information about the study protocol, confidential data management and they fill up a questionnaire before signing or refuse.

There are not inconveniences caused to the patients. No incentives are planned for the patients regarding the operation or the follow-up.

There are not any additional risks for the treatment and control group: both groups have the standard surgical treatment and follow-up: icodextrin group is an antiadherences fluid with no adverse effects.

All the medical information obtained from the patients are kept confidential among the research scientists conducting the study.

The patients are free to withdraw from the study, whenever they want and without any obligation.

The study is approved by the ethical Committee of the S'Orsola-Malpighi Hospital, Bologna, Italy

The primary endpoints of our study are:

To evaluate the therapeutic role of Icodextrin 4% to reduce ASIO incidence

To reduce adherences rate [in case of reoperation for ASIO]

The onset of any other complications are recorded intraoperatively, postoperatively, at discharge, at 7-days, 1-month, 6-months, every year up to 5 years follow-up.

The primary outcomes are assessed every year [interim analysis] up to 5 years after the last enrolled patient.

The data generated by this study are analysed in two ways. The continuous numerical data are subjected to analysis of variance [ANOVA], this method being applicable to the discrimination of two continuous populations. Discrete data are analysed by the chi-squared test or Fisher exact test, as appropriate. Statistically significant differences between study treatments are based on p < 0.05.

The study takes approximately 2 years for the inclusion period. According to the number of ASIO menaged monthly by each surgeon in our Department, the duration of the inclusion period can be approximately 2 years to reach the number of about 176 enrolled patients.

ASIO is a common disease. Any improvement in this field will benefit many patients reducing the re-operative rate.

## Competing interests

Each author has participated sufficiently in the work to take public responsibility for appropriate portions of the content. All authors read and approved the final manuscript and declare no competing interests.

## Authors' contributions

FC, LA have made substantial contributions to conception and design, or acquisition of data, or analysis and interpretation of data; AL, GE have been involved in drafting the manuscript or revising it critically for important intellectual content. ADP, LD conceived of the study, and participated in its design and coordination and helped to draft the manuscript.

## Supplementary Material

Additional file 1 **Ethical Committee Approval. Ethical Committee Approval.** Click here for file

## References

[B1] Ellis H (1997). The clinical significance of adhesion: focus on intestinal obstruction. Eur J Surg Suppl.

[B2] Menzies D (1993). Prospective adhesions: their treatment and relevance in clinical practice. Ann R Coll Surg Engl.

[B3] Ellis H (1998). The magnitude of adhesion-related problems. Ann Chir Gynaecol.

[B4] Fevang BT, Fevang J, Lie SA, Soreide O, Svanes K, Viste A (2004). Long-term prognosis after operation for adhesive small bowel obstruction. Ann Surg.

[B5] Ray NF, Denton WG, Thamer M, Henderson SC, Perry S (1998). Abdominal adhesiolysis: inpatient care and expenditures in the United States in 1994. J Am Coll Surg.

[B6] Brown CB, Luciano AA, Martin D, Peers E, Scrimgeour A, diZerega GS (2007). Adept [icodextrin 4% solution] reduces adhesions after laparoscopic surgery for adhesiolysis: a double-blind, randomized, controlled study. Fertil Steril.

[B7] Verco SJ, Peers EM, Brown CB, Rodgers KE, Roda N, diZerega G (2000). Development of a novel glucose polymer solution [icodextrin] for adhesion prevention: pre-clinical studies. Hum Reprod.

[B8] diZerega GS, Verco SJ, Young P, Kettel M, Kobak W, Martin D, Sanfilippo J, Peers EM, Scrimgeour A, Brown CB (2000). A randomized, controlled pilot study of the safety and e. cacy of 4% icodextrin solution in the reduction of adhesions following laparoscopic gynaecological surgery. Hum Reprod.

[B9] Schwartz SI, Spencer FC, Shires GT, eds (1999). Principles of surgery.

