# Supplementary material for: Prospective controlled randomized trial on prevention of postoperative abdominal adhesions by Icodextrin 4% solution after laparotomic operation for small bowel obstruction caused by adherences [POPA study: Prevention of Postoperative Adhesions on behalf of the World Society of Emergency Surgery]
Source: Trials. 2008 Dec 18;9:74. doi: 10.1186/1745-6215-9-74 (PMC2631497; doi:10.1186/1745-6215-9-74)
Supplement: Additional file 1 — Ethical Committee Approval. Ethical Committee Approval. [file 1745-6215-9-74-S1.pdf]

REGIONE EMILIA ROMAGNA
AZIENDA OSPEDALIERA DI BOLOGNA
Policlinico S. Orsola -Malpighi
Via Albertoni, 15- Tel. (051) 63.61.111 telefax (051) 63.61.202
40138 BOLOGNA
Casella Postale 2137- Cod. Fisc. 92038610371

Bologna 17th December 2006

IL DIRETTORE SANITARIO

The Hospital Manager

Chiar.mo Prof. Antonio D. Pinna
Direttore
U.O./ Chirurgia Trapianti di Fegato
e Multiorgano
Pad. 25 – S. Orsola
Policlinico S. Orsola-Malpighi

Oggetto: Sperimentazione Clinica 112/2006/U
Re: 112/2006/U POPA Study

In merito al parere espresso dal Comitato Etico, allegato alla presente,

According tho the Ethical Comittee approval

si autorizza

We authorize

l'attivazione dello studio in argomento.

This clinical study

Cordiali saluti.

Best Regards

Dr. Vito Bongiovanni
